# Mindfulness Mediates the Effect of a Psychological Online Intervention for Psychosis on Self-Reported Hallucinations: A Secondary Analysis of Voice Hearers From the EviBaS Trial

**DOI:** 10.3389/fpsyt.2020.00228

**Published:** 2020-04-03

**Authors:** Thies Lüdtke, Heike Platow-Kohlschein, Nina Rüegg, Thomas Berger, Steffen Moritz, Stefan Westermann

**Affiliations:** ^1^Department of Psychology, UiT – The Arctic University of Norway, Tromsø, Norway; ^2^Department of Psychiatry and Psychotherapy, University Medical Center Hamburg-Eppendorf, Hamburg, Germany; ^3^Department of Clinical Psychology and Psychotherapy, University of Bern, Bern, Switzerland

**Keywords:** mindfulness-based intervention, auditory verbal hallucinations, mediation analysis, schizophrenia, internet intervention

## Abstract

**Background:**

Psychological online interventions (POIs) could represent a promising approach to narrow the treatment gap in psychosis but it remains unclear whether improving mindfulness functions as a mechanism of change in POIs. For the present study, we examined if mindfulness mediates the effect of a comprehensive POI on distressing (auditory) hallucinations.

**Methods:**

We conducted a secondary analysis on voice hearers (*n* = 55) from a randomized controlled trial evaluating a POI for psychosis (EviBaS; trial registration NCT02974400, clinicaltrials.gov). The POI includes a module on mindfulness and we only considered POI participants in our analyses who completed the mindfulness module (*n* = 16).

**Results:**

Participants who completed the mindfulness module reported higher mindfulness (*p* = 0.015) and lower hallucinations (*p* = 0.001) at post assessment, compared to controls, but there was no effect on distress by voices (*p* = 0.598). Mindfulness mediated the POI’s effect on hallucinations (*b* = −1.618, LLCI = −3.747, ULCI = −0.054) but not on distress by voices (*b* = −0.057, LLCI = −0.640, ULCI = 0.915).

**Limitations and Discussion:**

Completion of the mindfulness module was not randomized. Hence, we cannot draw causal inferences. Even if we assumed causality, it remains unclear which contents of the POI could have resulted in increased mindfulness and reduced hallucinations, as participants completed other modules as well. In addition, confounding variables could explain the mediation and the sample size was small. Nonetheless, the overall pattern of results indicates that the POI is likely to improve mindfulness, and that increased mindfulness could partially explain the POI’s efficacy.

## Introduction

Approximately 40% of patients with psychosis do not receive treatment consistently (1). Psychological online interventions (POIs) could help to narrow this treatment gap. Barak et al. (2) describe such interventions as a “primarily self-guided intervention program that is executed by means of a prescriptive online program operated through a website and used by consumers seeking health- and mental-health related assistance” (p. 5). For depression and anxiety, meta-analyses indicate that POIs are effective (3, 4), so it seems promising to develop POIs for psychosis as well. So far, POIs for people with psychosis are scarce [e.g., (5)], but pilot studies and study protocols indicate that they are receiving increasing attention [e.g, HORYZON; (6, 7)].

POI approaches for psychosis differ in their scope to ameliorate psychotic symptoms and associated burden. While some interventions provide peer-to-peer networks or offer online platforms to share experiences (7, 8), another promising approach is to address potential psychological precursors of psychosis to alter psychotic symptoms indirectly. Studies have identified a variety of such precursors, mostly negative behavioral, cognitive, or affective states, such as sleep disturbances (9), worry (10), and depression (11). Theoretical models suggest that these variables are causal factors contributing to psychosis (12). There have been first attempts to address some precursors online [e.g., depression; (13)] but many other potential precursors have not yet received attention. One of them is mindfulness. Mindfulness could represent a functional coping strategy in psychosis that might be particularly effective in reducing the distress caused by auditory verbal hallucinations (AVHs) by promoting a nonjudgmental observation of sensory experiences.

Approximately three in four people with schizophrenia or schizoaffective disorder experience AVHs once in their life (14). Not only are AVHs common, they also cause considerable distress (15). From a cognitive behavioral perspective, AVHs reflect false external attributions of internal processes rather than purely perceptual phenomena (16), and beliefs about the voices cause negative affective consequences rather than their frequency. A person who hears voices twice as often does not necessarily suffer twice as much (17). Hence, cognitive behavioral therapy for psychosis (CBTp) aims at reducing distress and disturbance caused by voices rather than their frequency. Face-to-face CBTp has proven successful for the treatment of psychotic symptoms in general (18) and for voices specifically (19). Despite CBTp’s success, there is room for improvement in the psychological treatment of psychosis and AVHs in view of small effect sizes on overall positive symptoms (g = 0.16) in comparative trials (18). Mindfulness-based exercises could effectively add to the effects of CBTp as they provide patients with tools that go beyond the ones of CBTp and that could be particularly useful to reduce distress and disturbance caused by voices. Traditionally, CBTp aimed at identifying automatic thoughts and reevaluating them (20). This approach emphasized the importance of thoughts and their impact on feelings and actions. Mindfulness-based interventions, on the other hand, try to reduce a thought’s impact by not engaging with it at all. Instead of challenging a thought or a sensation, detached mindfulness helps to let such thoughts or experiences pass. So-called third-wave CBTp interventions illustrate how mindfulness-based exercises can complement CBTp (20).

Mindfulness is a diverse concept, which encompasses components such as decentering, awareness, and acceptance. As reviewed by Kabat-Zinn (21), mindfulness, which has its origins in Buddhist meditation techniques, can be subsumed as “moment-to-moment, nonjudgmental awareness”. Kabat-Zinn (22) shaped the definition of mindfulness as “paying attention in a particular way: on purpose, in the present moment, and nonjudgmentally” (p. 4). In the following, we refer to this definition of mindfulness. A common denominator of all mindfulness interventions is their goal to embrace present experiences in a nonjudgmental way without avoiding or suppressing them (23) thereby reducing distress, for example, elicited by AVHs (24). From a theoretical point of view, mindfulness helps people who experience AVHs to be aware of the sensation without letting the sensation define oneself. Basic research supports this notion: Mindfulness is negatively correlated with hallucinations and associated distress (25). Experiential avoidance (i.e., the attempt to avoid thoughts, feelings, memories or sensations) precedes psychotic symptoms in studies with longitudinal designs (26). Its counterpart, the mindfulness-based emotion regulation strategy “experiential acceptance,” appears to be superior to other emotion regulation strategies, such as reappraisal (24). In people with depression, mindfulness seems to be particularly effective at reducing worry and rumination (27), processes that are common precursors of psychosis (28).

Findings from “offline” treatment studies emphasize the potential of mindfulness-based interventions. A meta-analysis found that mindfulness-based interventions are effective at reducing hospitalization rates but also negative and affective symptoms, at a small to moderate effect size (23). A second meta-analysis encompassing both mindfulness and acceptance-based interventions found small to moderate short-term effects on total psychotic symptoms and positive symptoms, but not on negative symptoms of schizophrenia (29). The authors also report moderate evidence for lower hospitalization rates and shorter duration of hospitalization (29). Louise et al. (30) found no effect of mindfulness-based interventions on distress, positive, or negative symptoms of schizophrenia but only on depressive symptoms. Finally, participants perceive mindfulness-based interventions as safe and meaningful, leading to low dropout rates and high satisfaction (23, 25).

To date, it remains unclear whether the findings from mindfulness-based face-to-face interventions are transferable to internet-based interventions for psychosis. Furthermore, we do not know which role mindfulness plays in the effectiveness of CBT-based POIs in general, especially regarding AVHs. Possibly, improving mindfulness is an important mechanism of action in treating psychotic symptoms, such as AVHs. Considering the accumulating evidence for the effectiveness of mindfulness-based interventions and the possible benefits of online interventions for psychosis, we expect that POIs for psychosis with mindfulness components represent a promising approach. Hence, our group has developed a comprehensive CBT-based POI for psychosis, which encompasses a module on mindfulness [(31); Westermann et al.^1^1Westermann, S., Rüegg, N., Lüdtke, T., Moritz, S., & Berger, T. (under review). Internet-Based Self-Help for Psychosis: Findings from a Randomized Controlled Trial.]. While the POI covers the treatment of several putative precursors of psychotic symptoms, in this secondary paper, we focus on its effects on mindfulness, distressing AVHs, and general hallucinatory experiences. From a theoretical point of view, we expected that mindfulness is particularly effective at reducing distress associated with AVHs, so we included people reporting lifetime AVHs in our analyses. In addition, we only considered participants from the treatment group who used the mindfulness module of our POI. As our POI is not a purely mindfulness-based intervention, we cannot evaluate its mindfulness components directly, but we evaluate whether mindfulness acts as a mechanism of change. We hypothesized that (a) the overall POI reduces distress elicited by voices and (b) that changes of mindfulness mediate this effect.

## Methods

We conducted a secondary analysis on a subgroup of lifetime voice hearers obtained from the EviBaS trial [(31); Westermann et al.^1^]. The EviBaS trial is a preregistered (NCT02974400, clinicaltrials.gov) multicenter parallel group single-blind randomized controlled superiority trial with an allocation ratio of 1:1 comparing a waitlist control group to a POI for people with psychosis. The POI addresses persecutory delusions and AVHs, as well as presumed precursors of psychotic symptoms in web-based modules that participants can access via an internet browser. The POI includes modules on mindfulness (which we focus on in this paper), worry and rumination, social competence, self-worth, depression, sleep, and metacognitive biases, such as “jumping to conclusions” (32). An introductory module explains the rationale of the POI while a closing module provides information and worksheets on relapse. Each module contains both educational components as well as exercises, in which participants apply what they have learned to their own experiences. A smartphone application accompanies the POI and provides exercises for everyday life. Participants used their private computers and smartphones to access the POI and the application.

At baseline, participants completed an online assessment consisting of self-report questionnaires as well as a diagnostic interview via telephone conducted by trained personnel. When participants fulfilled eligibility criteria, we randomly allocated them to the waitlist control group or the intervention group using a web-based randomization tool (Random.org, RRID: SCR_008544). Participants from the intervention group received access to the POI for 8 weeks. After this period, all participants completed a post assessment, again consisting of a telephone interview and online self-report questionnaires. We did not provide any active treatment for the control group during the waiting period but one of the inclusion criteria was that all participants received either antipsychotics, psychotherapeutic treatment or (at least monthly) psychiatric consultations, or a combination of both. After the waiting period, participants from the waitlist group had access to the intervention, but this does not affect the data of this study.

**Figure 1:**
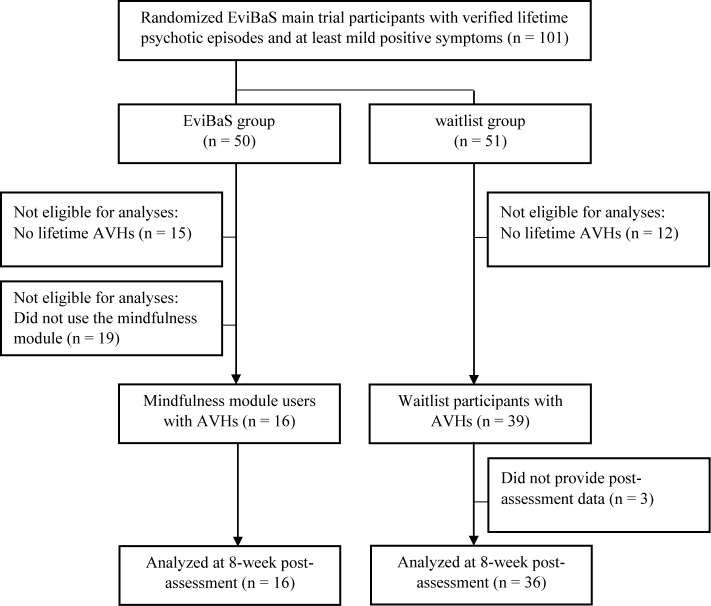
Flow chart depicting the selection of participants for the secondary analyses presented here.

Here, we report results from a subsample of the EviBaS trial consisting of participants who reported lifetime AVHs. In addition, we drew a subset from the participants allocated to the POI, namely those who, inter alia, used the mindfulness module (see **Figure 1** for a flow chart).

### Recruitment

The EviBaS trial took place in Germany and Switzerland. Local ethics committees have approved of the study (Cantonal Ethics Committee Bern, ID 03/14; German Society for Psychology, ID SM052015_CH). We recruited through a database of former participants with schizophrenia spectrum diagnoses. In addition, we advertised the study online and by contacting psychiatric institutions in Switzerland and Germany.

In accordance with the Declaration of Helsinki, participants gave informed consent prior to participation online. Eligibility criteria were an age of 18 years or older, access to the internet, sufficient command of the German language, a lifetime diagnosis of a nonaffective psychotic disorder (confirmed by trained study personnel in a diagnostic telephone interview), current positive symptoms of psychosis (delusions, suspiciousness, or hallucinations), and antipsychotic or psychotherapeutic treatment/psychiatric consultations (at least monthly), or both. We verified the diagnosis of a psychotic disorder as well as current positive symptoms in a diagnostic interview. We requested participants to fill in an emergency plan, a document listing contact persons that participants could reach out to in case of an emergency during their participation in the study. Exclusion criteria were acute suicidality, an acute danger towards others, or a diagnosis of a neurological disease of the central nervous system.

### The Mindfulness Intervention

**Figure 2:**
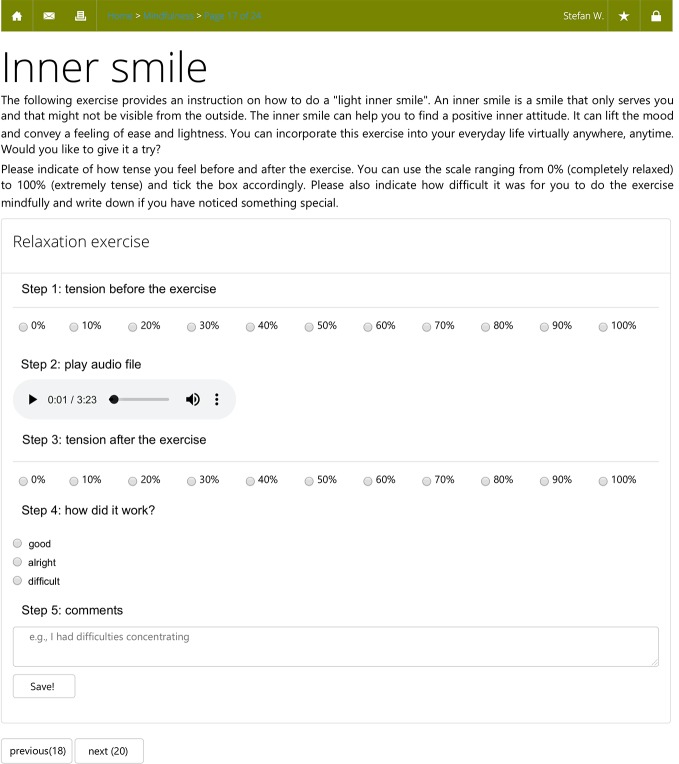
Translated web browser screenshot of the mindfulness intervention depicting the “inner smile” exercise.

Apart from an introductory module, which was mandatory, participants could decide whether they would like to work through a certain module of the POI or not. For this paper, we only analyzed participants who worked on the mindfulness module. **Figure 2** shows a screenshot from the mindfulness module. The mindfulness module consisted of 24 web pages, which contained text, pictures, and audio files. We made clear that the aim of the mindfulness module was to improve mindfulness and there was no cover story. The first 13 pages of the module provided psychoeducation on mindfulness, such as historical origins, effects of mindfulness on psychological health, as well as presumed associations with psychosis. The remaining 11 pages included mindfulness exercises, such as breathing exercises, the “S.T.O.P.” exercise (stop, take a mindful breath, observe what your feelings, thoughts etc., proceed your activity), and the “body scan” exercise. Participants received instructions via text, or via audio files, recorded by a psychotherapist. The whole mindfulness module took approximately 1 hour to complete. Participants could, however, repeat exercises if they wished to do so. Trained and supervised psychology students (“moderators”) guided participants throughout the POI. Moderators provided feedback once or twice per week via private messages and offered assistance.

### Measures

Rüegg et al. (31) provide a detailed description of all outcome measures included in the EviBaS trial. In the following, for the sake of brevity, we focus on outcomes relevant for the secondary analyses presented here. As in previous studies of our group [e.g., (33)], we report participants’ cumulated antipsychotic dosages instead of chlorpromazine equivalent values at baseline. The cumulated antipsychotic dosage indicates the percentage of the maximum dosage of a certain antipsychotic drug. We chose this metric because chlorpromazine equivalents have faced criticism regarding their validity for second generation antipsychotics (34) as their effectiveness appears to be related to different types of receptors instead of just dopamine (35). In addition, we hoped that the percentage of the maximum dosage would be easily accessible to the reader.

#### Psychopathology

We used the German version (36) of the Mini International Neuropsychiatric Interview [MINI; (37)] to verify the diagnosis of a nonaffective psychotic episode, as well as comorbid diagnoses, via telephone. The MINI is a structured interview with good specificity (37). To assess psychotic symptom severity, we used the Positive and Negative Syndrome Scale [PANSS; (38)]. The PANSS measures positive, negative, and global symptoms of schizophrenia on 30 items rated on 7-point scales. Higher scores reflect more severe symptoms. The PANSS shows good psychometric properties (39). Participants were only eligible to participate if they received a score of three or higher on at least one of the items P1 (delusions), P3 (hallucinations), or P6 (suspiciousness/persecutory delusions).

#### Mindfulness

We measured mindfulness using the Mindful Attention and Awareness Scale [MAAS; (40)]. On 6-point Likert scales, the MAAS measures participants’ ability to mindfully experience the current moment. The German version of the MAAS shows a good internal consistency of α = 0.83, good test-retest reliability of *r* = 0.82, and correlations with subjective well-being (41). Higher scores reflect more mindfulness.

#### AVHs

We used a subset of items from the Delusion and Voices Self-Assessment (DV-SA; 42) to measure self-reported distress caused by AVHs. We calculated the self-generated subscale “distress by voices” consisting of the items “distress”, “obedience”, “control”, “interference with relationships”, and “interference with activities”. Higher scores reflect more severe symptoms. The original voices scale shows good internal consistency of α = 0.83. Test-retest reliability ranged from 0.86 to 0.96 (42). To our knowledge, there is no German version of the DV-SA. Our group translated the scale for the EviBaS trial. We chose the respective items of the distress by voices subscale based on theoretical considerations. With α = 0.83, the internal consistency of the newly created “distress by voices” subscale was good in our sample (*n* = 55). Because conclusions based on a nonvalidated subscale of the DV-SA are limited, we included another measure of hallucinations as well, which is well established and validated. The German adaption (43) of the Launay Slade Hallucination Scale Revised [LSHS-R; (44)] shows good internal consistency in the general population (α = 0.83) and in patients with psychosis (α = 0.87). On 12 items, the LSHS-R measures both subclinical as well as pathological hallucinatory experiences on 5-point Likert scales. Higher scores reflect more severe symptoms.

### Statistical Analyses

We used SPSS 25^®^ (SPSS, RRID: SCR_002865) for all analyses. For the mediation analysis, we used the PROCESS^®^ macro provided by Andrew Hayes (45). All significance tests were two-sided with a significance level of *p* = 0.05. We report effect sizes as η^2^_p_ with η^2^_p_ = 0.01 as small, η^2^_p_ = 0.06 as medium, and η^2^_p_ = 0.14 as large effects. We compared groups at baseline using *t*-tests and χ^2^-tests. We conducted ANCOVAs to examine baseline-corrected posttreatment group effects of mindfulness, distress by voices, and hallucinations. Apart from baseline scores, ANCOVAs did not include additional covariates. Post hoc, however, we repeated analyses with gender as an additional covariate to account for unequal gender distributions. To answer the question whether mindfulness functions as a mechanism of change, we conducted a mediation analysis with group allocation as the independent variable, posttreatment distress by voices as the outcome, pre-post change scores of mindfulness as the mediator, and baseline distress by voices as a covariate. We repeated the analysis with LSHS-R hallucination scores instead of distress by voices. For PROCESS^®^ analyses, we report robust bootstrap confidence intervals based on a resampling procedure with 5,000 samples (LLCI = lower level confidence interval, ULCI = upper level confidence interval). We report complete cases analyses, which include participants who completed the post assessment of respective outcomes (*n* = 52; 95%).

## Results

### Retention, Adherence, and Baseline Characteristics

Between December 2016 and May 2018, we recruited a sample of *n* = 101 participants in the EviBaS trial, which was below the targeted sample size of 140 (based on a power calculation assuming an at least medium-sized effect in the main trial; see Westermann et al.^1^). Possibly, the extensive and hence demanding baseline assessment impeded the recruitment process. Out of *n* = 7,237 persons who visited a study website to obtain information about the trial, *n* = 746 gave informed consent to participate and began to complete the online assessment. In total, *n* = 140 potential participants finished the baseline questionnaires as well as the telephone interview and were diagnosed with a schizophrenia-spectrum disorder, of which *n* = 101 participants fulfilled the inclusion criterion of at least mild symptoms on the PANSS items P1, P3, or P6 (see Westermann et al.^1^). Missing the target sample size led to reduced power. This applies particularly to the secondary analyses presented here, given that we only analyzed a subgroup of participants. For the analyses presented here, we drew a subset of participants who reported lifetime AVHs and who used the mindfulness module, if allocated to the POI (54%). That is, we excluded POI participants from analyses, if they did not use the mindfulness module. Consequently, the group sizes of the POI group (*n* = 16) and the waitlist group (*n* = 39) are unequal. **Table 1** displays sample characteristics. The distribution of women and men differed significantly between groups, with a higher proportion of women in the POI group compared to the waitlist group. With PANSS total scores of roughly 65, our sample could be described as mildly to moderately ill (46). We present adherence for the mindfulness module only (for information on overall adherence to the POI, see Westermann et al.^1^). The mean time spent in the module was 74 min (*SD* = 53 min). The distribution was skewed with one person spending much time in the mindfulness module (256 min), as indicated by a lower median of 61 min.

**Table 1:** Baseline characteristics.

Characteristics	waitlist (*n* = 39)	POI (*n* = 16)	Statistics
Demographics			
Age in years, mean (SD)	41.36 (9.25)	41.69 (9.88)	*t* (53) = 0.12, *p* = 0.907
Education in years, mean (SD)	11.59 (1.77)	11.69 (1.30)	*t* (53) = 0.20, *p* = 0.843
Gender, proportion female (%)	18/39 (46%)	13/16 (81%)	χ^2^ (1) = 5.68, *p* = 0.017*
Clinical variables			
PANSS total, mean (SD)	67.10 (15.45)	61.63 (19.16)	*t* (53) = 1.11, *p* = 0.271
PANSS positive, mean (SD)	15.36 (4.93)	14.88 (4.81)	*t* (53) = 0.33, *p* = 0.741
PANSS negative, mean (SD)	12.62 (4.35)	11.00 (4.62)	*t* (53) = 1.23, *p* = 0.225
PANSS global, mean (SD)	29.08 (7.16)	26.69 (8.87)	*t* (53) = 1.05, *p* = 0.300
MINI: current major depressive episode (%)	12/39 (31%)	6/16 (38%)	χ^2^ (1) = 0.23, *p* = 0.629
MINI: current psychotic disorder (%)	24/39 (62%)	11/16 (69%)	χ^2^ (1) = 0.26, *p* = 0.614
Taking antipsychotics (%)	34/39 (87%)	11/16 (69%)	χ^2^ (1) = 2.59, *p* = 0.108
Cumulated antipsychotic dosage, mean (SD)	46.50 (38.41)	36.13 (44.58)	*t* (50) = 0.86, *p* = 0.396
Completing post assessment online (%)	35/39 (90%)	16/16 (100%)	χ^2^ (1) = 1.77, *p* = 0.183
Outcome variables at baseline			
DV-SA distress by voices, mean (SD)	3.92 (3.94)	3.31 (4.13)	*t* (53) = 0.52, *p* = 0.609
MAAS mindfulness, mean (SD)	3.84 (0.91)	3.50 (1.12)	*t* (53) = 1.18, *p* = 0.244
LSHS-R hallucinations	17.97 (11.35)	20.69 (12.35)	*t* (53) = 0.79, *p* = 0.436

Cumulated antipsychotic dosage = The sum of the dosages of a participant’s antipsychotic drugs divided by the maximum dosage of each drug; e.g., if the maximum dosage of a drug is 20 mg per day and a participant takes 10 mg of that drug, this equals a score of 50%. MINI, Mini International Neuropsychiatric Interview. Distress by voices = sum score of items 4, 7, 8, 9, and 10 from the Delusion and Voices Self-Assessment (DV-SA) questionnaire. The cumulated antipsychotic dosage was not available for all participants, hence the lower df. *p < 0.05.

### Effects of Group Allocation on Mindfulness, Distress by Voices, and Hallucinations

We examined assumptions before all analyses. According to visual inspection, the assumption of normality within groups was violated for distress by voices due to several low scores and hence a skewed distribution. All other assumptions were met. We conducted three separate ANCOVAs examining the group effect on distress by voices (DV-SA-subscale), mindfulness (MAAS), and hallucinations (LSHS-R) at postassessment (see **Table 2**). Group allocation did not influence distress by voices. Relative to controls, posttreatment mindfulness was significantly higher in the POI group, while LSHS-R hallucinations were significantly lower. In exploratory complete cases analyses, we compared waitlist participants to intervention group participants who did not use the mindfulness module (*n* = 19), and found no effects (all *p*’s > 0.478).

**Table 2:** Complete cases ANCOVAs showing the baseline corrected effect of group allocation on distress by voices, mindfulness, and Launay Slade Hallucination Scale Revised (LSHS-R) hallucinations (*n* = 52).

Outcome	Adjusted means waitlist (SE)	Adjusted means POI (SE)	Complete cases ANCOVAs
Distress by voices	3.38 (0.38)	3.02 (0.58)	*F* (1; 49) = 0.281, *p* = 0.598, η^2^_p_ = 0.006
Mindfulness	3.83 (0.10)	4.28 (0.15)	*F* (1; 49) = 6.346, *p* = 0.015*, η^2^_p_ = 0.115
Hallucinations	16.78 (1.09)	9.56 (1.64)	*F* (1; 49) = 13.360, *p* = 0.001*, η^2^_p_ = 0.214

POI, psychological online intervention; all ANCOVA models include the baseline values of the respective outcome as covariates. *p < 0.05

### Mindfulness as a Mediator of Group Differences

Group allocation did not influence distress by voices at postassessment. Nonetheless, we conducted the mediation analysis to identify a possible suppression effect (47). However, there was no mediation. The group effect on distress by voices remained nonsignificant (direct effect: *b* = −0.309, SE = 0.756, *t* = 0.409, *p* = 0.685). The nonexistent mediation was confirmed by the bootstrap confidence interval of the indirect effect (indirect effect: *b* = −0.057, LLCI = −0.640, ULCI = 0.915).

As there was a significant positive effect of the intervention on LSHS-R hallucinations, we examined whether increased mindfulness would mediate this effect, which was the case. Adding mindfulness change scores as a mediator reduced the group difference of posttreatment hallucinations but it remained significant (direct effect: *b* = −5.600, SE = 2.052, *t* = 2.729, *p* = 0.009). The bootstrap confidence interval of the indirect effect confirmed a significant mediation (indirect effect: *b* = −1.618, LLCI = −3.747, ULCI = −0.054). The unstandardized coefficient of the indirect effect indicates that mindfulness accounted for 1.618 points of the group difference in LSHS-R score at posttreatment. Because of the unexpected baseline group differences regarding gender (i.e., the proportion of women was higher in the POI group compared to the waitlist group), we decided to repeat the analyses with gender as a covariate. We found that the direction and significance of effects remained unchanged.

## Discussion

We conducted a secondary analysis with a subsample from the EviBaS trial [(31); Westermann et al.^1^], consisting of people with psychosis who reported lifetime AVHs. As our focus was on mindfulness, we only analyzed POI participants who used the mindfulness module. We hypothesized that the POI and its mindfulness components would lead to reduced distress by voices and increased mindfulness at postassessment when compared to a waitlist control condition. In addition, we hypothesized that an increase of mindfulness would mediate the effect on distress by voices. To account for methodological concerns regarding the self-generated and nonvalidated distress by voices scale, we also examined the group effect on hallucinations measured with the LSHS-R and conducted the mediation analysis accordingly.

Contrary to our expectations, distress by voices at postassessment did not differ between groups. However, LSHS-R hallucinations and self-reported mindfulness differed between groups at postassessment, both in favor of the POI group. There was a significant mediation of the group effect on posttreatment hallucinations. Increased mindfulness explained a significant proportion of the group difference in posttreatment hallucination scores. After adjusting for the mediator, the direct effect remained significant. Therefore, mindfulness explains the effect only partly.

We did not evaluate a purely mindfulness-based intervention. Although we only analyzed data from participants who used the mindfulness module, those participants also used other modules of the intervention. Hence, we cannot draw causal conclusions regarding the effect of the mindfulness-exercises within our comprehensive POI for psychosis. Nonetheless, the pattern of results indicates that mindfulness functions as a mechanism of change in our POI. Firstly, the mediation analysis showed that an increase of mindfulness accounted for a significant portion of the group effect on hallucinations. This result indicates that the POI partly reduced hallucinations by increasing mindfulness. Secondly, at post assessment, POI participants reported higher self-reported mindfulness compared to a waitlist control condition. Considered separately, each individual result suffers from methodological limitations (see limitations). Taken together, however, the consistent pattern of results indicates that mindfulness played an important role in the effectiveness of the POI, despite the fact that our design does not allow causal conclusions.

We chose the outcome “distress by voices” based on theoretical considerations. Although we examined a sample of people with AVHs, many participants did not experience stress elicited by voices and hence, there was little to no room for improvement for most participants. The broader outcome “hallucinatory experiences” measured with the validated LSHS-R scale, however, captured experiences of a much larger proportion of participants, making it more suitable as an outcome measure. Due to the methodological concerns of the “distress by voices” scale, we argue that our study does not allow drawing definite conclusions regarding the POIs effect on distress by voices. The finding does coincide with a previous study, though. In a randomized controlled trial conducted by Gottlieb et al. (5), the web-based program “coping with voices” resulted in significantly greater increases of social functioning compared to usual care but there was no effect on the severity of auditory hallucinations (5). There are important differences between our study and the one conducted by Gottlieb et al. (5), such as a slightly different outcome (clinician rated auditory hallucinations vs. self-reported hallucination-associated stress) or the strength of the control condition (waitlist vs. usual outpatient care). Nonetheless, the results are comparable and indicate no effect of web-based interventions/POIs on AVHs specifically.

As described above, the main reason to include the LSHS-R scale was to account for methodological shortcomings of our self-generated “distress by voices” scale. From a theoretical point of view, however, we would have expected effects on distress, only. The effect on overall hallucinatory experiences was surprising but could partly be due to properties of the LSHS-R scale. Firstly, the scale measures experiences that are present in the general population (48). Hence, the items were “easier”, which resulted in more room for improvement. Secondly, and more importantly, several items from the scale measure experiences, which show large overlap with mindfulness-related processes. For example, the first item of the scale captures difficulties in concentrating, which can be interpreted as a lack of mindfulness: “No matter how hard I try to concentrate, unrelated thoughts always creep into my mind” (48). Our mindfulness module specifically aimed at improving a person’s ability to mindfully experience the current moment without distractions.

### Limitations and Future Directions

Firstly, we did not evaluate a purely mindfulness-based but a comprehensive POI, which addresses mindfulness among other factors. Hence, we cannot conclude that the specific module was responsible for the positive effects on mindfulness or hallucinations. We excluded participants who did not use the mindfulness module but our participants did not use the mindfulness module exclusively. It is unlikely that administering the mindfulness module as a stand-alone intervention would have yielded similar results. From a therapist’s point of view, however, increased mindfulness and reduced hallucination severity are desirable outcomes, irrespective of which exercise of the POI accounted for it. Secondly, usage of the mindfulness module was nonrandomized and adherence low with almost 50% of the intervention group not using the mindfulness module. As completion of the mindfulness module depended on participants’ own preference, it is likely that a selection bias resulted in a highly motivated and hence not representative subsample of participants. This also becomes apparent in a significantly higher proportion of female participants in the subgroup completing the mindfulness module. Female gender is associated with higher adherence in online interventions (49). Controlling for gender, however, did not affect results in our analyses. Analyzing only the subgroup of participants who completed the mindfulness module resulted in a selective and a small POI sample (*n* = 16). Statistical analyses with small sample sizes are accompanied by low power, which might partly explain why we did not find effects on distress by voices. At the same time, small sample sizes increase the risk of overestimating effect sizes, which might have affected the significant group differences. Thirdly, we relied on self-report measures instead of clinician-rated scales to measure mindfulness. Self-report measures have faced severe criticism in mindfulness research (50). The same criticism applies to the measurement of voice hearing and associated distress. A clinician-rated instrument, such as the psychotic symptom rating scales [PSYRATS; (51)], would have led to findings that are more durable. Fourthly, the mediation analysis does not allow concluding that increased mindfulness causally led to a reduction of hallucinations. Possibly, a third variable explains the mediation, such as mindfulness-associated affect: Mindfulness predicts positive affect in psychosis (52), while negative affect predicts psychotic symptoms, such as paranoia (53). Therefore, it is possible that not mindfulness but associated affective changes accounted for the effects in our study. Fifthly, participants were aware that the intervention aimed at improving mindfulness. Positive effects on self-reported mindfulness could hence reflect a social desirability bias. Finally, as mentioned before, findings based on the distress by voices scale are questionable. We did not validate the scale *a priori* and the distribution of scores was not normal in groups, limiting the informative value of the ANCOVA.

Despite the methodological concerns of this secondary analysis, our results indicate that mindfulness-based exercises complement CBT-based POIs effectively. Even if we cannot examine the unequivocal contribution of the POI's mindfulness exercises, our mediation analysis indicates that adding mindfulness exercises to CBT-based POI’s is a promising approach. In addition, participants with psychosis wish for the treatment of a broad range of treatment targets other than positive symptoms of psychosis (54) and mindfulness-based interventions are well accepted (23). Consequently, we argue that POIs for psychosis could benefit from adding mindfulness exercises in the treatment of hallucinations and that mindfulness represents a worthwhile outcome in POI studies. However, our secondary analysis does not allow causal conclusions, as usage of the mindfulness module was voluntary and not randomized. In order to draw causal inferences about mindfulness-based POIs on voice related stress, we need further randomized controlled trials evaluating purely mindfulness-based POIs with appropriate clinician-rated outcome measures. In addition, the small sample size limits the reliability of the estimated effects. Hence, there is a need for sufficiently powered future studies to replicate our preliminary findings.

## Data Availability Statement

The datasets generated for this study are available on request to the corresponding author.

## Ethics Statement

The studies involving human participants were reviewed and approved by Kantonale Ethikkommission für die Forschung, Murtenstrasse 31, 3010 Bern, Switzerland and Ethik-Kommission der DGPs, Universität Trier, Fachbereich I Psychologie, D-54286 Trier, Germany. The ethics committee waived the requirement of written informed consent for participation.

## Author Contributions

TL and HP-K contributed equally to the development of the design, data acquisition, data analysis, interpretation of the data, and drafting of the manuscript. SW, SM, and NR contributed to the design, data acquisition, data analysis, and data interpretation. They critically revised the manuscript, and provided important intellectual input. TB critically revised the manuscript, provided important, intellectual input, contributed to the development of the design, and data acquisition. All authors approve of the content of this manuscript and agree to be accountable for all aspects of the work in ensuring that questions related to the accuracy or integrity of any part of the work are appropriately investigated and resolved.

## Funding

The EviBaS trial received funding from the Swiss National Science Foundation (project number 159384) and the German Research Foundation (project number DFG Mo 969/17–1). This secondary analysis did not receive additional funding.

## Conflict of Interest

The authors declare that the research was conducted in the absence of any commercial or financial relationships that could be construed as a potential conflict of interest.
